# The Nijmegen Decision Tool for Chronic Low Back Pain. Development of a Clinical Decision Tool for Secondary or Tertiary Spine Care Specialists

**DOI:** 10.1371/journal.pone.0104226

**Published:** 2014-08-18

**Authors:** Miranda L. van Hooff, Jan van Loon, Jacques van Limbeek, Marinus de Kleuver

**Affiliations:** 1 Sint Maartenskliniek, Department of Research, Nijmegen, The Netherlands; 2 Sint Maartenskliniek, Department of Orthopedics, Nijmegen, The Netherlands; 3 Achmea Health Insurance Company, Zeist, The Netherlands; 4 VU University Medical Center, Department of Orthopedics, Amsterdam, The Netherlands; University of Hong Kong, Hong Kong

## Abstract

**Background:**

In Western Europe, low back pain has the greatest burden of all diseases. When back pain persists, different medical specialists are involved and a lack of consensus exists among these specialists for medical decision-making in Chronic Low Back Pain (CLBP).

**Objective:**

To develop a decision tool for secondary or tertiary spine care specialists to decide which patients with CLBP should be seen by a spine surgeon or by other non-surgical medical specialists.

**Methods:**

A Delphi study was performed to identify indicators predicting the outcome of interventions. In the preparatory stage evidence from international guidelines and literature were summarized. Eligible studies were reviews and longitudinal studies. Inclusion criteria: surgical or non-surgical interventions and persistence of complaints, CLBP-patients aged 18–65 years, reported baseline measures of predictive indicators, and one or more reported outcomes had to assess functional status, quality of life, pain intensity, employment status or a composite score. Subsequently, a three-round Delphi procedure, to reach consensus on candidate indicators, was performed among a multidisciplinary panel of 29 CLBP-professionals (>five years CLBP-experience). The pre-set threshold for general agreement was ≥70%. The final indicator set was used to develop a clinical decision tool.

**Results:**

A draft list with 53 candidate indicators (38 with conclusive evidence and 15 with inconclusive evidence) was included for the Delphi study. Consensus was reached to include 47 indicators. A first version of the decision tool was developed, consisting of a web-based screening questionnaire and a provisional decision algorithm.

**Conclusions:**

This is the first clinical decision tool based on current scientific evidence and formal multidisciplinary consensus that helps referring the patient for consultation to a spine surgeon or a non-surgical spine care specialist. We expect that this tool considerably helps in clinical decision-making spine care, thereby improving efficient use of scarce sources and the outcomes of spinal interventions.

## Introduction

In Western Europe, Low back pain (LBP) is considered to have the greatest burden of disease for society [1]. In this global burden of disease study LBP is ranked higher than for example cancer, heart disease, cerebrovascular disease, chronic obstructive pulmonary disease and asthma, osteoarthritis or diabetes. In the Netherlands, approximately 44% of the population experiences at least once an episode of LBP, with one in five reporting persistent back pain resulting in chronic low back pain (CLBP; LBP lasting for more than three months [2], [3]) with substantial limitations in functional activities after one year [4], [5]. As the prevalence of CLBP appears to be increasing [6], CLBP is not only a burden for the patient but the related healthcare costs and productivity due to absence of work have a high health and socioeconomic impact on western societies [7]–[9]. Not surprisingly, CLBP is among the most common complaints of patients visiting a medical specialist in secondary care, i.e. spine surgeons, physiatrists, rheumatologists, pain consultants. The high number of CLBP patients overwhelms these healthcare providers and a significant number of second opinions and re-interventions are evident. With the limited health care budgets and given the high prevalence of CLBP and its substantial socioeconomically impact, it is essential to use resources of healthcare providers efficiently and to triage CLBP patients adequately in order to make sure that these patients see the right care giver timely. However, as yet such a valid classification system or decision tool is lacking and secondary care medical specialists are failing to reliably identify which patients will benefit from which surgical or non-surgical intervention.

One challenge in the development of a decision tool is that the CLBP population is heterogeneous. Therefore, it is unlikely that one intervention benefits all [10]. A longstanding duration of complaints is the only one common defining feature. It makes CLBP a complex problem and in fact it is a symptom referring to the location of the problem rather than a specific diagnosis [11]. The term itself is non-diagnostic for an underlying pathology and lacks specificity. Many authors have emphasized the biospychosocial influences on the development CLBP and persistence of symptoms [12], [13] and a broad multidimensional approach is widely recognised. However, the failure to differentiate between underlying causes is one of the reasons that various surgical and non-surgical interventions exist for the same problem [14]. Moreover, studies evaluating these interventions for CLBP have led to inconsistent results [3], [15]–[19] and rarely show more than a small to moderate overall benefit [3], [20], [21].

It is suggested that several different CLBP patient profiles might be identified which are likely to benefit from different recommended interventions [1], [18], [19], [22]–[25]. These profiles are based on indicators modifying the effects of interventions [26] and with that related to the outcomes [24]. The ultimate outcomes of spinal interventions are patients' improved quality of life, restored functional status and relieved pain [27]. However, due to methodological heterogeneity, the current evidence is inconclusive regarding predictive indicators for a successful treatment outcome. Even though it is recognized that CLBP ‘without biological causes’ has to be distinguished from other spinal disorders that respond reliably to surgery [25], [28], a recently performed nationwide survey among Dutch spine surgeons showed that even in the group ‘with presumed biological causes’ a lack of consensus exists in surgical decision making [29]. To distinguish patient profiles several treatment outcome-based classifications for decision making exist. However, they are all developed and studied as a guide for non-surgical interventions applied in primary care [24]. As a challenge with probably the greatest potential for improving outcomes and efficiently guiding patients to the right secondary health care professional (e.g. spine surgeon, pain consultant, physiatrist, rheumatologist), it is recommended to develop a classification system to direct CLBP patients, presented in secondary or tertiary back care, to both surgical and non-surgical interventions, based on biomedical and psychosocial indicators [11], [22]–[25], [30], [31].

Therefore, the purpose of this study is to develop a clinical decision tool for CLBP, based on evidence in international guidelines and literature, and expert panel consensus using indicators predicting a successful treatment outcome. The decision tool supports secondary or tertiary back care specialists to decide which patients should be considered for a surgical intervention and which patients for a non-surgical intervention and therefore, it aims to triage patients to the appropriate health care professional. The ultimate goal is to improve treatment outcomes and to reduce related costs for society.

## Methods

**Figure 1:**
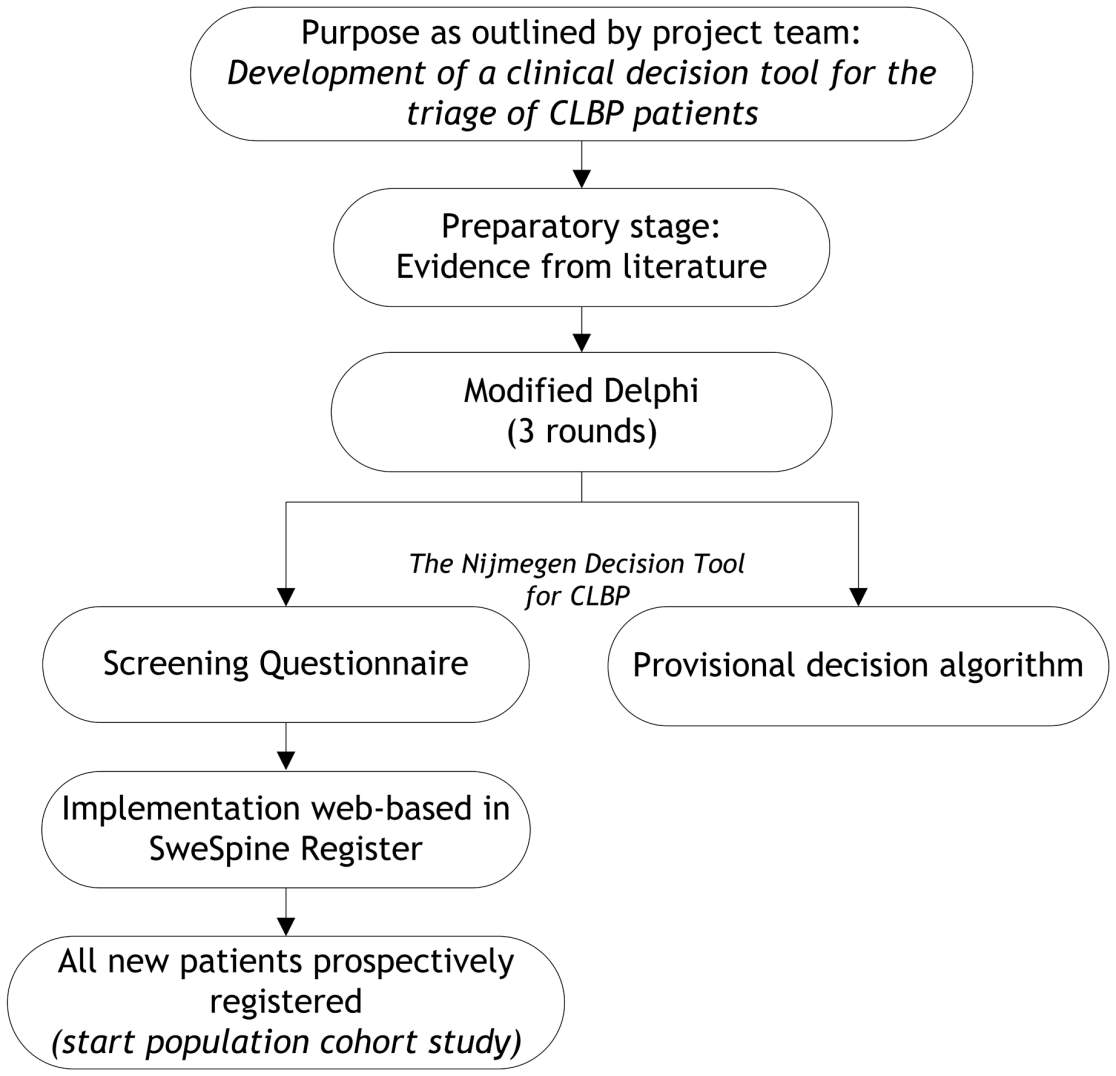
Flow diagram of the development of the Nijmegen Decision Tool for CLBP.

This study aimed to identify indicators predicting the outcome of interventions and the persistence of CLBP complaints by two stages: a preparatory stage followed by a three-round Delphi study. The preparatory stage consisted of a literature review. As we expected inconclusive evidence in the literature, a formal consensus (Delphi) procedure among a heterogeneous panel of experts in the CLBP field was planned and performed. We used a Modified Delphi Technique in order to realise an optimal integration of research-based knowledge and the clinical experience of experts [32] on this topic. Having identified the predictive indicators, a clinical decision tool, including a screening questionnaire and a provisional decision algorithm, was compiled. In the flow diagram of Figure 1 the overall process of the development of the Nijmegen decision tool for CLBP is presented.

### Preparatory stage: Evidence from literature

The indicator set from which a clinical decision tool can be constructed is based on evidence found in international guidelines and in the literature, as these guidelines are normative for evidence based daily practice. As a starting point, the clinical flag approach [33] for clinical decision-making in CLBP and the indicators as recommended in the guidelines [22], [23], [34], are used. We performed a literature review searching for indicators predicting outcome of invasive or non-invasive interventions and persistence of CLBP. Appropriate studies were traced using MedLine, EMBASE and the Cochrane Library. The most relevant used search terms were: ‘back pain’ [MesH], ‘chronic’, ‘predict’, ‘prognosis’ [MesH], ‘persistent’, ‘treatment outcome’ [MesH], ‘rehabilitation’ [MesH], ‘surgery’ [MesH]. The search was restricted to include systematic and narrative reviews, randomized controlled trials (RCT) and prospective cohort studies. Studies were included when 1) CLBP was the primary complaint; 2) published in the period 2000–2010; 3) involved either surgical interventions for CLBP or non-surgical interventions or persistence of CLBP complaints; 4) age between 18–65 years; 5) baseline measures of predictive indicators are reported, as the time of assessment may influence the prognostic value of treatment outcome [35], [36]; 6) at least one of the reported outcome measures had to assess functional status, quality of life, pain intensity, employment status or a composite score. CLBP was defined as more than three months continual or recurrent episodes of LBP [2], [3].

There were no language restrictions. Moreover, reference lists of included articles were scrutinized to identify articles not captured in the database search. When a systematic review was included, the original longitudinal studies (RCT or observational) of that systematic review were excluded from the current sample to avoid duplication or double use of the same data.

We used four international guidelines [22], [23], [31] and one national guideline [34]. The literature search revealed 33 relevant papers: eight systematic reviews [36]–[43], four narrative reviews [44]–[47], three randomized studies [48]–[50], and eighteen observational studies [51]–[68]. All potential predictive indicators were classified into five main domains: sociodemographic; pain; somatic; psychological; and functioning & quality of life. Of each paper, data of available evidence was extracted regarding the predictive values of measured baseline determinants (indicators). The evidence is weighed according to the Levels of Evidence as defined by the Oxford Centre for Evidence-Based Medicine [69]. Per indicator the evidence is categorized into four categories: 1) indicator with proven predictive value (PV; evidence found that the concerning indicator has predictive value), 2) indicator with proven no predictive value (NP; evidence found that the concerning indicator has no predictive value), 3) indicator with inconclusive evidence (I; conflicting evidence found), and 4) indicator with no evidence found in literature (N). Subsequently, all indicators (PV, NP, and I) were selected and used for Phase 2 of this study (the Delphi Study). Indicators with non-predictive value and categorized NP were excluded from the sample as the evidence showed no predictive value for treatment outcome or for persistence of CLBP complaints. The included indicators are summarized in an evidence table, according to the design used in the related studies (data available in Table S1). Per indicator the evidence was summarized: the evidence is conclusive and of predictive value (C: PV) or the evidence is conclusive and of no predictive value (C: NP) or inconclusive evidence (I). These results are used in the Delphi study (Delphi-1 & 2).

### Delphi Study

The Delphi technique is a commonly used method to develop clinical guidelines [70] and also used in healthcare indicator research [71]. The technique was originally developed in the 1950s by Dalkey and Helmer at the RAND Corporation as a method of eliciting and refining group judgements [72]. Delphi may be characterized as a systematic method for structuring a group communication process so that the process is effective in allowing a group of experts or ‘expert panel’, as a whole, to deal with a complex problem [73]. The method relies on three key features: 1) anonymous response to guarantee equality in experts opinions, 2) iteration and controlled feedback, and 3) statistical analysis of group responses [72]. The Delphi technique, as recently described [32], [71], is a structured process that uses a recommended series of two or three rounds to gather expert opinions. When reaching consensus is difficult or consensus is unclear a physical panel meeting at the end is recommended, under the condition that the meeting should be well structured and should take place in favourable conditions (surrounding and environment) with a moderator (process leader), who is not one of the panellists, to contain the influence of dominant personalities (Modified Delphi Technique).

#### Project team

A project team was formed to conduct the process and the research and comprised a methodologist who is also a physician and who has a background in statistics (JvL), an orthopaedic spine surgeon (MdK) and a health scientist (MvH). The responsibilities of this project team were performing a review of clinical predictive indicators, weighing the evidence for each indicator, selecting a panel of experts, developing the questionnaires, organisation and conduct of email rounds and consensus meeting, analysing the responses, and compiling a draft version of the clinical decision tool.

#### Panellists

In the area of CLBP treatment different medical specialists are involved and knowledge gaps exist between different medical specialties [24]. Therefore, a heterogeneous group of experts was selected for the expert panel. Moreover, it is known that when exploring areas of uncertainty, a heterogeneous group is appropriate [70] and it is expected that heterogeneity in a decision-making group may lead to better performance [71]. Panellists were asked based on their willingness to participate, their intention to commit to the process, and their recognised knowledge of the topic. They were recruited in one hospital and were included in the panel when they met the following criteria: (1) professional background as a orthopaedic spine surgeon, anaesthesiologist & pain consultant, physiatrist, rheumatologist, psychologist, physical therapist, occupational therapist, psychomotor therapist, nurse practitioner; (2) CLBP care and cure is the main area of professional attention; (3) more than five years of clinical experience in the field; and (4) ability and willingness to respond to each email Delphi round within one week and to join the final Delphi consensus meeting.

#### Delphi procedure

The panellists were emailed explaining the purpose and the content of the study. To increase participation the panellists were asked to reply if they were willing to join and whether they intended to commit to the procedure. The whole procedure was performed in two months, April–May 2011. Two email Delphi rounds were planned to reach consensus. Consensus was defined as a ‘general agreement of a substantial majority’. The threshold for general agreement was set at ≥70%. If an indicator reached a second time disagreement, the indicator is rejected. For the two email rounds participants were asked to respond and reply within one week. A third round, the final consensus meeting was performed to reach consensus about the included items and to construct a first draft clinical decision tool. During this meeting participants were allowed to discuss issues and exchange views supported by evidence, with the aim to resolve issues for indicators that had not passed the threshold for consensus. In each round the purpose and procedure of the current Delphi round and following Delphi rounds were explained.

#### Delphi-1

The initial draft list of indicators extracted from the literature review and arranged in a conceptual framework of domains was provided to the expert panel. They were asked to respond to three main sets of questions (Q). The Q1 set was based on international and national guidelines, which recommend an assessment of a minimal set of consistent prognostic indicators influencing the treatment outcome [22], [23], [31], [74]. Compiled by the project team and supported by the literature review, this minimal set consisted of 32 indicators (‘red’ and ‘yellow’ flags, expectation of recovery, socio-economic status, sick leave, pain severity, prior episodes of LBP), for which agreement (YES or NO) was asked. The Q2 set was based on the results of the literature review and included 26 indicators with weighted evidence for which agreement for inclusion (YES or NO) was asked. Moreover, in Q3 the panellists were given the opportunity to suggest additional indicators for inclusion, based on scientific evidence and provided to the project team, and to write general comments. The items for which ≥70% agreement was reached were selected and included in the draft list for the final Consensus meeting (Delphi-3). The indicators for which consensus was not reached, were included in round 2 (Delphi-2).

#### Delphi-2

In the second round an anonymous feedback report with a summary of results of Delphi-1 was provided. In this summary an overview of results for each question and each indicator was given in count and percentages of agreement. Moreover, all suggested and newly formulated indicators, including the arguments and comments were presented. The Delphi-2 questionnaire contained both those indicators that did not reach the pre-set agreement level of ≥70% (Q1 and Q2; Delphi-1) and those that were newly formulated by the panellists (Q3; Delphi-1). In this round the panellists were requested to indicate with YES or NO which of the indicators of Q1 and Q2 absolutely needed to be included in the list? In Q3 a possibility was given to mention new indicators. The level of agreement was set at ≥70% among the panellists, i.e. these indicators were selected and included in the draft list for the following Consensus meeting (Delphi-3). A second time lack of consensus led to rejection of the concerning indicator.

#### Delphi-3 Consensus meeting

Before the meeting all panellists received a covering summary of results on both Delphi rounds which was similarly described and drafted as for the results of the first round. Moreover, a draft list was provided with indicators for which consensus (≥70%), no consensus (<70%) was reached, and the rejected indicators. During the meeting all indicators for which previously no consensus had been reached were reconsidered. Only if new arguments based on scientific or clinical evidence were provided, an attempt to reach a new consensus on that item was made. Moreover, the panellists were encouraged to consider alternative views when consensus could not be achieved [70]. The meeting had a formal character to ensure that all panellists had a chance to express their views, all indicators were considered, no discussion was allowed and only arguments could be provided, and the panellists made judgements individually. Consensus was reached by voting; raising hands. Only those indicators with ≥70% agreement were included in the final screening questionnaire, all others were rejected. A dedicated and independent process leader is a key element for a successful consensus meeting; this person facilitates the exchange of relevant information [70]. One of the project team members (JvL) is an experienced Delphi round facilitator, who was not one of the panellists, but who ensured that the process ran smoothly and that good-quality un-biased decisions were made. The project leader (MvH), not a member of the expert panel, assisted the process leader in process monitoring, ensured that all procedures ran according to the rules, counted the votes, compiled the minutes during the meeting and provided a full report after the meeting. The report included the followed procedures, the results of the voting rounds, the course of the discussions, the decisions made, and the final list of ‘consensus indicators’. All panellists who joined the consensus meeting received a copy.

### Development of the ‘Nijmegen decision tool for CLBP’

The final list of ‘consensus indicators’ was used to compile a first version of the clinical decision tool. For the screening questionnaire existing international patient reported outcome measures (PROMs) were screened to identify whether the indicators are covered by these PROMs. The indicators were compared to existing questions used in the Swedish Spine Register (Swespine [www.4S.nu]). These questions were translated and screened for unambiguity and whether they measured the construct as intended by the indicator. The remaining indicators were converted to new questions. The screening questionnaire was built in the Dutch patient interface of Swespine. Based on the list of consensus indicators, international guidelines, and current practice a provisional decision algorithm was constructed.

## Results

### Preparatory stage: Evidence from literature

An initial draft list with 58 candidate indicators, categorized in five domains, and including the evidence was compiled. Table 1 shows the evidence summarized for all candidate indicators (the evidence and references per indicator are available in Table S1). For 38 (66%) candidate indicators conclusive evidence was found indicating a predictive value for treatment outcome or persistence of complaints (C: PV), 15 had inconclusive evidence for predicting outcome or persistence of pain complaints (I), and for five indicators conclusive evidence was found that the concerning indicator is of no predictive value (C: NP). These five indicators were removed from the initial draft list, leaving 53 candidate indicators and they were included in the Delphi study.

**Table 1:** Results Preparatory stage: Evidence from literature.

Domain	Category^1^	Study design^2^				Evidence^3^
		*SR*	*RCT*	*PC*	*NR*	
		n	n	n	n	
**Sociodemographic**	**Personal**					
	Age	4	-	8	4	I
	Gender	4	-	9	3	I
	Ethnicity	1	-	-	-	C: PV
	Body weight	3	-	1	-	I
	Marital status†	3	-	-	2	C: NP
	**Health**					
	Smoking	5	-	4	1	I
	Previous back surgery	1	-	2	-	C:PV
	Use of analgesics	1	-	1	1	I
	**Social**					
	Education	1	-	3	3	I
	Social status	1	-	-	1	I
	Functioning – leisure	1	-	1	-	I
	Social support	-	-	-	2	C: PV
	**Work**					
	Socio-economic status*	2	-	2	2	C: PV
	Work satisfaction	5	-	-	4	I
	Functioning – work	3	-	-	-	I
	Sick leave*	3	-	2	2	C: PV
	Compensation	3	-	3	-	I
	Litigation	-	-	-	1	C: PV
	Work ability	1	-	-	-	C: PV
	Work adjustment	1	-	-	-	C: PV
	Physical strenuousness	1	-	-	-	I
**Pain**	Duration	4	-	3	1	I
	Intensity*	5	1	11	3	C: PV
	Intensity – back	-	-	3	-	C: PV
	Intensity – leg	2	-	2	-	C: PV
	Interference daily activities	2	-	-	1	C: PV
	Frequency/preceding (prior) episodes*	1	-	1	2	I
**Somatic**	Diagnosis; co morbidities	1	-	2	1	C: PV
Physical & Biological	Bulging or protruded disc*	2	-	2	-	C: PV
	Loss of neurological function*	1	-	1	1	C: PV
	Red flags (n = 10)* #					C: PV
	Strength; endurance; mobility†	4	-	2	-	C: NP
	Central sensitisation†	1	-	-	-	C: NP
	Postural control; psychomotor speed†	1	-	-	-	C: NP
**Psychologic**	**Psychic affect**					
	Distress*	6	2	3	4	C: PV
	Anxiety*	3	2	2	4	C: PV
	**Cognition**					C: PV
	Catastrophizing*	5	3	7	3	C: PV
	Somatization*	4	-	1	1	C: PV
	Coping*	5	-	2	4	C: PV
	Intelligence†	1	-	-	-	C: NP
	**Behaviour**					
	Fear of movement/(re)injury*	3	-	12	4	C: PV
	Expectations – work return*	1	-	3	2	C: PV
	Expectations – outcome/recovery*	2	-	1	2	C: PV
	Self-efficacy (incl. Readiness–to–change)	-	-	1	-	C: PV
	Pain avoidance & pain persistence	-	1	-	-	C: PV
**Functioning &**	Functioning in daily activities & walking	6	1	9	3	C: PV
**Quality of Life**	Health-related physical functioning	3	-	5	1	C: PV
	Health-related mental functioning	6	-	1	1	I
	General perceived health	1	-	2	1	C: PV

Initial draft list with indicators (n = 58) indicating a predictive value for treatment outcome or persistence of back pain, categorized in domains, including the number of studies found per study design and the resulting evidential value.

1. * Recommended in (inter-/) national guidelines;

# Pain started age <20 or >50 years, recent trauma, constant progressive pain, history of malignancies, prolonged use of corticosteroid use, HIV, recent unexplained weight loss, structural deformity, infectious disease (CBO 2010).

2 . *SR* Systematic Review; *R* Randomized Clinical Trial; *PC* Prospective Cohort study; *NR* Narrative Review; *n* number of studies.

3 . *I* Inconclusive evidence; *C* Conclusive evidence; *PV* Predictive Value; *NP* No Predictive value.

4. † Not included for phase 2 Delphi Study.

### Delphi Study

#### Participants

A panel of 29 experts met the inclusion criteria and agreed to participate (orthopaedic spine surgeon [n = 7], anaesthesiologist & pain consultant [n = 3], physiatrist [n = 3], rheumatologist [n = 1], psychologist [n = 4], physical therapist [n = 7], occupational therapist [n = 1], psychomotor therapist [n = 1], nurse practitioner [n = 2]).

The response rate for the first Delphi round (Delphi-1) was 76% (n = 22) and for Delphi-2 69% (n = 20). The main reason for not responding in the first two rounds was due to absence from work and none of the approached panellists did not respond on both email rounds. All 29 panellists (100%) attended the final consensus meeting (Delphi-3).

#### Delphi-1

**Figure 2:**
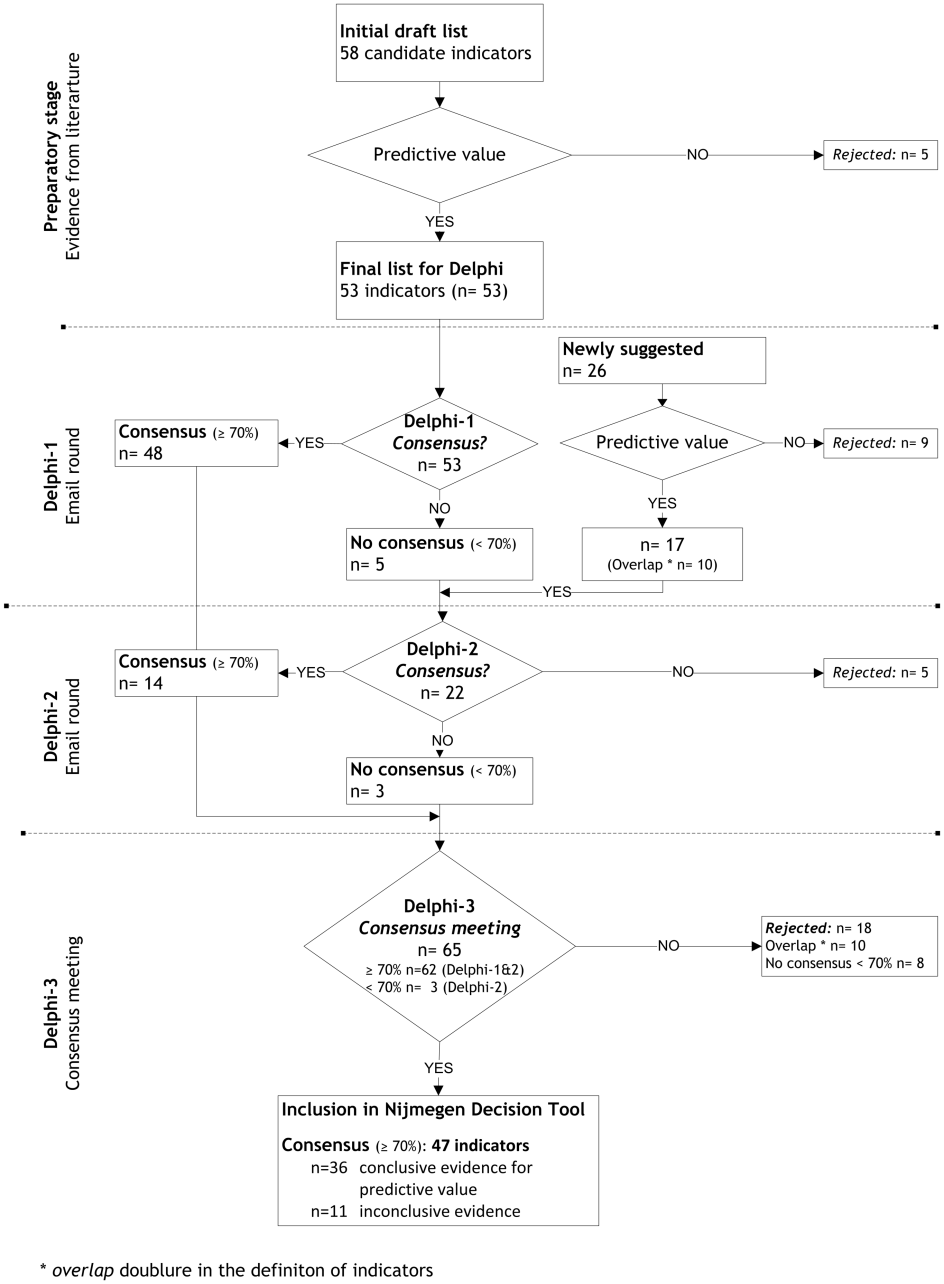
Results of the Delphi Study. At each stage of the Delphi study the expert panel consensus for presented indicators is shown. To reach consensus the level of agreement was set at ≥70%. Indicators reaching full consensus were included in the Nijmegen Decision Tool.

As shown in Figure 2, 48 indicators were selected in the first round based on consensus (≥70% agreement level). For five indicators consensus was not reached. Moreover, 26 indicators were newly formulated by the panel in the open end question (sociodemographic n = 9; pain n = 4; somatic n = 7; psychologic n = 3; functioning and quality of life n = 3) and these indicators were added to the Delphi-2 questionnaire. These 26 indicators consisted of:

- six indicators mentioned in the Dutch guidelines for general practitioners: self-management of complaints, previous interventions, daily course of pain complaints, influence of rest, mobility and posture, previous episodes, and comorbidities (range and severity) [75]. The last two show overlap with previously identified indicators.
- one indicator for inflammatory LBP (Calin criteria [76]).
- ten newly formulated indicators with overlap with indicators of the initial draft list.
- nine indicators with no predictive evidence and were rejected from the sample.

No further comments were made.

#### Delphi-2

As shown in Figure 2, 22 indicators were presented, including the 17 newly formulated and the five indicators for which no consensus was reached in Delphi-1. Of these, 14 reached the pre-set ≥70% agreement level for consensus. Five indicators were rejected as for the second time no consensus was reached. No new indicators were suggested and no further comments were made.

#### Delphi-3 Consensus meeting

All indicators on which consensus were reached in either Delphi-1 or 2 (62 indicators; 48+14) and those indicators no consensus was reached in Delphi-2 (3 indicators) were briefly discussed (Figure 2). After each indicator the panellists voted whether they still agreed or not. As shown in Table 2, at the meeting consensus was reached for 47 indicators (pre-set ≥70% agreement level), whereas eight indicators reached no consensus. These indicators were rejected, as well as ten indicators showing an overlap with the initial indicators.

**Table 2:** Results of the Delphi Study.

Domain	Category	Evidence
**Sociodemographic**	**Personal**	
	Age	I
	Gender	I
	Body weight & BMI	I
	**Health**	
	Smoking	I
	Previous back surgery	C
	Use of analgesics	I
	*Self-management of complaints*	C
	*Interventions in the past*	C
	**Social**	
	Social status	I
	Functioning – leisure	I
	Social support	C
	**Work**	
	Socio-economic status*	C
	Work satisfaction	I
	Functioning – work	I
	Sick leave*	C
	Litigation	C
**Pain**	Duration	I
	Intensity*	C
	Intensity – back	C
	Intensity – leg	C
	Frequency/preceding (prior) episodes*	I
	*Daily course of pain complaints*	C
	*Influence of rest, mobility, and posture*	C
**Somatic**	Diagnosis; co morbidities (Red Flag)	C
Physical & Biological	Bulging or protruded disc* (Red Flag)	C
	Loss of neurological function* (Red Flag)	C
	Red flags (n = 11)*	C
	Pain started age <20 or >50 years	
	Significant trauma	
	Pain is constant and non-mechanical	
	Pain in thoracic spine	
	Deformities (i.e. scoliosis, lumbar kyphosis)	
	Previous history of malignities/cancer	
	History of intravenous drug use	
	AIDS/HIV	
	Currently steroid use	
	Recent unexplained weight loss	
	*Calin criteria for axial spondylarthritis*	C
**Psychologic**	**Psychic affect**	
	Distress*	C
	Anxiety*	C
	**Cognition**	
	Catastrophizing*	C
	Somatization*	C
	Coping*	C
	**Behaviour**	
	Fear of movement/(re)injury*	C
	Expectations – work return*	C
	Expectations – outcome/recovery*	C
**Functioning &**	Functioning in daily activities & walking	C
**Quality of Life**	Pain-interference daily activities	
	Health-related physical functioning	C

* Recommended in international guidelines.

Newly formulated indicators are printed in italics.

*I* Inconclusive evidence; *C* Conclusive evidence.

Final list with ‘full consensus’ indicators categorized in domains, including the resulting evidential value.

Consensus was reached to re-formulate one indicator ‘Body weight’ into ‘Body weight & BMI’ and another indicator ‘Pain-interference daily activities’ switched domains from ‘Pain’ to the domain ‘Functioning & Quality of Life’.

The remaining 47 indicators formed the backbone of the screenings questionnaire (36 with conclusive evidence for predictive value [77%] and 11 with inconclusive evidence; Table 2).

### The ‘Nijmegen decision tool for CLBP’

A first version of a clinical decision tool consisting of two parts was drafted by the project team: (1) A screening questionnaire, including all 47 indicators, and (2) a provisional decision algorithm.

#### 1. The screening questionnaire

For the backbone of the screening questionnaire existing international patient reported outcome measures (PROMs) with well-established psychometric properties were screened to identify whether the 47 identified indicators were covered by these existing questionnaires (Table 3). Four of the 47 indicators are outcome indicators and adequately measured by the Oswestry Disability Index (ODI, version 2.1a) for functional status, the Short-Form 36 Health Survey Questionnaire (SF36) and the EuroQol (EQ-5D) for quality of life, and the Numeric Rating Scale (NRS) for back and leg pain. The STarT back is used as a screening tool for identifying the amount of risk for three psychological indicators (distress, catastrophizing, and fear of movement/(re)injury; i.e. ‘yellow flags’). The remaining 40 indicators were compared to existing questions in the Swespine register. Analogous questions were translated and the remaining indicators were added as dichotomous or multiple choice questions in the final questionnaire. The complete screening questionnaire is available from the authors.

**Table 3:** Backbone screening questionnaire.

Domains	Flag approach ^33^	Results Phase 2 *(current study)* Indicators (n = 47)	Questions
**Sociodemographic**	Blue & Black	13	Multiple choice
		3	Dichotomous
**Pain**	n.a.	5	Multiple choice
		2	NRS (0–10)*
**Somatic**	Red	14	Dichotomous
**Psychosocial**	Yellow	3	STarT back screening tool^88^
		4	Multiple choice
		1	Dichotomous
**Functioning & Quality of Life**	n.a.	2	ODI (v2.1a); SF36; EQ-5D*

* standard & agreed to implement in Swespine register www.4s.nu.

#### 2. The provisional decision algorithm

The provisional decision algorithm is based on the flag approach and based on current practice. The red flag signs are thought to be associated with underlying pathology. Therefore, in the algorithm the presence of ≥1 red flag (e.g. previous history of malignancies, trauma) is indicative for a consultation by a spinal surgeon, whereas a high risk on yellow flags (i.e. distress, catastrophizing cognitions) is second most decisive as a high risk on yellow flags might be predictive for treatment failure.

## Discussion

The purpose of this study was to develop a clinical decision tool for secondary or tertiary care specialists to decide which patients with chronic low back pain (CLBP) should be seen by a spine surgeon for consideration of a beneficial surgical intervention (including invasive pain management), and which patients in the future should best be seen by other medical specialists, e.g. physiatrists, rheumatologists or pain consultants. A study, consisting of a preparatory stage in which evidence from literature was summarized followed by a three-round Delphi study, contributed to the developed Nijmegen clinical decision tool for CLBP, which includes 1) a patient-based and web-based screening questionnaire and 2) a provisional decision algorithm.

In the preparatory stage of this study, we included in the literature review the evidence found in international guidelines [22], [23], [31] and the evidence from one national guideline [34], as these guidelines are normative for evidence based daily practice. However, studies included in these guidelines have led to inconsistent results and rarely show more than small to moderate overall benefit for different types of interventions, which makes it difficult to interpret which patient benefits from which intervention. Therefore, we performed a literature search covering the whole spectrum of CLBP ignoring specific medical specialties (explicit knowledge). This is supplemented by professional state-of-the-art knowledge derived from experiences in daily practice and collegial meetings and conferences, in a formal consensus (Delphi) study (implicit knowledge).

### 1. The screening questionnaire

The literature search revealed a large number of published studies (n = 33) related to the identification of predictive indicators for a successful treatment outcome or the prediction of persistence of CLBP complaints. As expected the result of this study is a long list of predictive indicators (n = 47), with most of them (77%) having scientific evidence for predictive value. To list and classify the indicators in Table 2 and 3 we used the conceptual model of patient outcomes (Poolman 2009) and identified five main domains: Sociodemographic, Pain, Somatic, Psychologic, and Functioning and Quality of Life. Overall, we found strong predictive evidence for successful outcome of spinal surgery for: previous back surgery and biological indicators (i.e. diagnosis; co-morbidities as diabetes, bulging or protruded disc, loss of neurological function, and ‘red flags’). In this study consensus was reached to add BMI and smoking as indicators in the screening questionnaire and to evaluate their contribution to outcome of surgical interventions over time. Although the evidence is growing that high BMI [77] and smoking [63], [64], [78]–[80] are predictive for a poor outcome after surgery, the current scientific evidence is still inconclusive. Along with the predictive value of psychological indicators (yellow flags) [38], [39], [48] and expectations for treatment outcome [37], [43], [78] and work return [37], [43], [56], predictive indicators as high disability [36], [38], [39], [48], [80], being unemployed [37], [43], [64], and being involved in litigation and/or compensation claims [36], [39], [41], [51], [63], [66], [78] seem to lead to unfavourable outcome for all CLBP interventions.

### 2. The provisional decision algorithm

For this study we used the recommended clinical flag approach [33] for clinical decision-making in CLBP as a starting point [22], [23], [74]. A diagnostic triage based on ‘red flag’ signs is recommended [22], [74], [81] as red flag signs are features thought to be associated with a high risk of serious underlying disorders, such as infection, inflammatory disease, cancer or fracture [33], [82] or nerve root disease [46]. The presence of a red flag alerts clinicians to the need for further examination and specific management [82]–[84]. In this study consensus was reached that the presence of one or more red flag signs is indicative for a consultation by a spinal surgeon, which was incorporated as a first step in the provisional decision algorithm. However, the guideline recommendations on diagnostic triage based on red flags are still not very strong [81]. Most of the patients with back pain show at least one positive red flag and do not have a serious underlying condition. Taking the guideline recommendations literally could cause harm. These harms include unnecessary diagnostics, unnecessary exposure to radiation, as well as unnecessary treatments, including surgery [85]. Moreover, a summary [86] of two recently published Cochrane reviews aiming to detect the diagnostic accuracy of red flags to screen for vertebral fracture [84] and malignancy [83] concluded that a lack of evidence exists that one red flag used in isolation can be used to aid a clinician's judgement. We expect that combinations of red flags and clinical features might appear more informative to assist clinical decision-making [83], [84], [86]. Even though it is recommended to assess the so-called yellow flags [22], [23] as well, it remains unclear what these indicators contribute to actual clinical decision-making. Large prospective studies are needed to evaluate the contribution of these indicators to successful treatment outcome. In this study consensus was reached that a high risk on yellow flags is the second most decisive for surgical or non-surgical interventions. We currently perform further studies to examine multifactorial diagnostic models and with that, the scientific value of combinations of flags and indicators, collected by means of the screening questionnaire, in clinical decision-making for further diagnostics and/or treatment.

CLBP is a multifactorial health condition and therefore, it has been widely recommended to develop a classification system or a decision tool to direct CLBP patients to interventions based on biomedical and psychosocial indicators [11], [22]–[25], [30], [31]. To date we are not aware of any study covering the whole spectrum and to our knowledge the Nijmegen decision tool for CLBP is the first published patient screening questionnaire and provisional decision algorithm. The backbone of the screening questionnaire consists of Dutch versions of international validated PROMs. To be able to make our future study results comparable and to be able to perform benchmark studies in the future, we selected commonly used PROMs covering those indicators that are treatment outcome-related (functioning in daily activities with ODI, quality of life with SF36 and EQ5D, and pain intensity with NRS) [87]. These PROMs are also used in the Swedish Spine Register (Swespine [www.4S.nu]). To screen yellow flags and determine the risk of psychological influence on treatment outcome we implemented the Dutch version of the STarT back screening tool [88]–[90]. Although validated and useful in primary care [88], [91]–[93] further research is needed to evaluate the validity and feasibility of prognostic screening with this tool in secondary or tertiary back care. To our knowledge, for the remaining indicators of the screening questionnaire no validated and reliable questionnaires exist. Large and methodological sound studies are needed for the feasibility and validity of these questions and whether (a combination of) these indicators contribute to successful treatment outcome.

In March 2012 the screening questionnaire was implemented in the Dutch patient interface of the Swespine. Swespine was chosen as it is one of the largest, oldest and most studied national registries, which covers both PROMs and clinical results [94], which allows benchmarking data in future. After pilot testing and some minor adjustments (e.g. grammatical and spelling mistakes, wording of questions, and technical issues related to the system), the web-based register started in May 2012. The registry is an ideal instrument to obtain meaningful data prospectively, to define normative values, to identify patient profiles, to confirm differences in treatment outcomes for subpopulations [25], [95]. The results can be used for quality assurance, quality improvement and for research purposes [94]. To study the provisional clinical decision algorithm, since May 2012 all LBP patients referred to our clinic complete the screening questionnaire web-based and treated patients are systematically followed over time for two years by completing the same PROMs at predefined follow-up moments. With that, in future it should be possible to identify patient profiles (phenotypes} predicting a beneficial treatment outcome for each type of surgical or non-surgical intervention, for all the referred, treated and untreated patients. At the same time data of the individual patients are presented in PDF-format in the electronic medical record (EMR) of the patient and contributes to individual decision-making in the clinic.

### Strengths and Weaknesses

Although for 36 of the 47 indicators conclusive evidence is available in the literature that they have predictive value for treatment outcome in patients with CLBP, for 11 indicators the evidence was inconclusive. These indicators were included in the Delphi study, based on the expert opinion of a panel of LBP clinicians. However, the formal, structured, and systematic character of the Modified Delphi Technique is of great value in indicator research when scientific evidence for indicators is inconclusive or lacking [71]. Moreover, to overcome the knowledge gaps existing between different medical specialties in the CLBP field [24], we used this technique in a multidisciplinary panel of specialists as it is argued to successfully bring together and to synthesize the knowledge of the whole expert group [71]. All health professionals came from one hospital specialised in spine care and the generalisation to other secondary or tertiary spine practices in other countries and healthcare environments might be limited. Strength of this study is that the panel included diverse professionals covering the secondary surgical and non-surgical CLBP care. Moreover, the decision tool is based on international accepted guidelines and evidence published in literature, covering the whole spectrum of CLBP. We weighed the evidence in literature according to the Levels of Evidence [69]. By combining this explicit knowledge with the implicit knowledge of the expert panel in the three-round Delphi study, after refinement of the decision algorithm, and after validation of the tool in other settings, we expect that the Nijmegen Decision Tool for CLBP could be used in general secondary and tertiary spine care.

### Conclusion

This study has provided the first clinical decision tool for CLBP patients, based on current scientific evidence and formal consensus, covering the whole spectrum of CLBP. We expect that this relatively simple tool will considerably help a daily spine practice in clinical decision-making 1) to select the right CLBP patients for the right interventions, thereby improving the outcomes of spinal interventions, and 2) lead to a reduction in healthcare costs by reducing the number of inappropriate referrals to spine care professionals.

## Supporting Information

Table S1 **Preparatory stage - Summarized evidence and References per indicator.**
(PDF)
