# Supplementary material for: The Nijmegen Decision Tool for Chronic Low Back Pain. Development of a Clinical Decision Tool for Secondary or Tertiary Spine Care Specialists
Source: PLoS One. 2014 Aug 18;9(8):e104226. doi: 10.1371/journal.pone.0104226 (PMC4136789; doi:10.1371/journal.pone.0104226)
Supplement: Table S1 — Preparatory stage - Summarized evidence and References per indicator. (PDF) [file pone.0104226.s001.pdf]

Supporting Information Table S1: Preparatory stage - Summarized evidence and References per indicator

DOMAIN	INDICATORS	EVIDENCE<br>Persistence LBP	Surgical	Non-surgical	*Overall (weighted)*	REFERENCES<br>Persistence LBP	Surgical	Non-surgical
**Sociodemographic**	***Personal***							
	Age	**I**	**I**	**NP**	**I (S)**	NP [41,44,47,60-61,67]; PV [45-46,52,66]	NP [38,63]; PV [39,43,55,65]	NP [36]; PV [66]
	Gender	**I**	**I**	**NP**	**I (S)**	NP [41,44,47,52,58,60-61,67]; PV [45,66]	NP [55,65]; PV [39,43,51,56]	NP [36]
	Marital status	**NP**	**NP**	**N**	**NP (S)**	NP [52,67]	NP [38,43,65]	
	Body weight	**I**	**NP**	**NP**	**I (S)**	NP [41](weight); I [46](weight)	NP [43](weight),[63](weight)	NP [36]
	Ethnicity	**PV**	**N**	**N**	**PV (W)**	PV [67]		
	Health							
	Smoking	**I**	**I**	**NP**	**I (S)**	NP [41,53]; PV [46]	NP [38,43]; PV [51,63-64]; I [39]	NP [36}
	Previous back surgery	**N**	**PV**	**N**	**PV (S)**		PV [51,64]; I [39]	
	Use of analgesics	**PV**	**N**	**I**	**I (S)**	PV [53]		I [36]
	Social							
	Education	**PV**	**I**	**N**	**I (S)**	NP [46]; PV [45,47,52,53]; I [66]	NP [64]; PV [38]	
	Social status	**PV**	**PV**	**NP**	**I (S)**	PV [47]		NP [36]
	Functioning - leisure	**PV**	**N**	**PV**	**I (S)**	PV [52]		PV [36]
	Social support	**PV**	**PV**	**N**	**PV (W)**	PV [44-45]		
	Work							
	Socio-economic status *	**PV**	**PV**	**N**	**PV (S)**	NP [53,67]; PV [37,46-47]	PV [43,64]	
	Work abiltity	**N**	**N**	**PV**	**PV (S)**			PV [36]
	Work satisfaction	**I**	**I**	**PV**	**I (S)**	NP [41,45,47]; PV [37,44,46]	PV [38]; I [39]	PV [36]
	Functioning - work	**PV**	**PV**	**PV**	**I (S)**	PV [41]	PV [38]	PV [36]
	Work adjustment	**N**	**N**	**PV**	**PV (S)**			PV [36]
	Physical strenuousness	**N**	**NP**	**N**	**NP (S)**		NP [43]	
	Sick leave *	**PV**	**PV**	**I**	**PV (S)**	PV [44,47,53]	NP [43]; PV [38,45]	I [36}
	Compensation	**PV**	**I**	**I**	**I (S)**	PV [41]	PV [51, 63]; I [39}	PV [66]; I [36]
	Litigation	**PV**	**N**	**N**	**PV (W)**	PV [46]		
**Pain**	Duration	**PV**	**PV**	**NP**	**I (S)**	NP [61,66]; PV [44,52]	PV [38-39,43]	NP [36]
	Intensity *	**PV**	**PV**	**PV**	**PV (S)**	PV [41,44,46-47,52-53,57-62,66]	PV [38-39,51,55,65]	PV [36,40,48]
	Intensity - back	**N**	**PV**	**PV**	**PV (W)**		PV [51,56]	PV [66]
	Intensity - leg	**I**	**PV**	**PV**	**PV (S)**	NP [47]; PV [41]	PV [56]	PV [36,66]
	Interference daily activities	**PV**	**PV**	**PV**	**PV (S)**	PV [47](bothersomeness)		PV [36]
	Frequency / preceding (prior) episodes *	**I**	**N**	**N**	**I (S)**	NP [41,52]; PV [44,46]		
**Somatic**<br>***Physical & Biological***	Strength, endurance, mobility	**I**	**I**	**NP**	**NP (S)**	NP [41]; PV [53]	NP [55]; I [39]	NP [36,40]
	Centralization phenomenon	**N**	**N**	**NP**	**NP (S)**			NP [36]
	Postural control, psychomotor speed	**N**	**N**	**NP**	**NP (S)**			NP [36]
	Diagnosis; comorbidities *	**I**	**PV**	**NP**	**PV (S)**	NP [66]; PV [44]	PV [43](comorbidity),[65](comorbidity)	NP [66]
	Bulging or protruded disc *	**PV**	**PV**	**N**	**PV (S)**	PV [41,52]	PV [38]	
	Loss of neurological function *	**PV**	**PV**	**PV**	**PV (S)**	PV [46]	PV [38]	PV [66]
	*Red Flags (n= 10) **	**N**	**PV**	**PV**	**PV (S)**			
**Psychologic**	***Psychic affect***							
	Distress *	**PV**	**PV**	**PV**	**PV (S)**	NP [67]; PV [42,44-47,49-50,53]	PV [38-39,43,65]	PV [40]; I [36]
	Anxiety *	**PV**	**PV**	**PV**	**PV (S)**	NP [47]; PV [42,44-46; 49-50,53,66]	PV [38-39]	PV [66]
	Cognition							
	Catastrophizing *	**PV**	**PV**	**PV**	**PV (S)**	PV [41-42,44-45,47,49-50,54,58,60-62,66]	PV [39,56]	PV [40,48]
	Somatization *	**PV**	**PV**	**N**	**PV (S)**	NP [66]; PV [41-42,45]	PV [38-39]	
	Intelligence	**N**	**N**	**I**	**I (S)**			I [36]
	Coping *	**PV**	**PV**	**PV**	**PV (S)**	NP [47]; PV [41-42,44-46]	PV [38-39,55]	PV [36,40]
	Behaviour							
	Self-efficacy (including Readiness to Chang	**PV**	**N**	**N**	**PV (W)**	PV [59](self-efficacy)		
	Fear of Movement / (re)injury *	**PV**	**PV**	**PV**	**PV (W)**	PV [41-42,44-47(pain),49,52,54,57-62,66]	PV [55-56]	PV [40,66]
	Avoidance & persistence	**PV**	**PV**	**N**	**PV (W)**	PV [50]		
	Expectations - work return *	**PV**	**I**	**N**	**PV (S)**	PV [37,44-45]	NP [55]; PV [56]	
	Expectations - outcome / recovery *	**PV**	**PV**	**N**	**PV (S)**	PV [37,53]	PV [43,55]	
**Functioning**<br>**& Quality of Life**	Functioning in daily activities & walking	**PV**	**PV**	**PV**	**PV (S)**	NP [67]; PV [41,44,46-47,52-53]	PV [38-39,51,55-56,63](functioning),[43,65](walking)	PV [36,40,48], [66](walking]
	Health (related physical QoL)	**I**	**PV**	**PV**	**PV (S)**	NP [53,67]; PV [41,45]	PV [43,56,63-64]	PV [36]
	Health (related mental QoL)	**I**	**PV**	**N**	**I (S)**	NP [53]; PV [41,45]	PV [43,51,56,63-64]	
	General perceived health	**N**	**PV**	**N**	**PV (W)**	NP [41]; PV [53]	PV [56]	

* International Guidelines [22,23,31]	**PV**= Predictive value<br>**NP**= No predictive value	**(W) weak** = studies or narrative reviews<br>**(S) strong** = studies & reviews or systematic reviews	***Refences used:***<br>Systematic reviews [36-43]<br>Narrative reviews [44-47]
**OUT**	**I** = Inconclusive evidence		Randomised studies [48-50]
**IN**	**N** = No evidence available		Observational studies [51-68]
